# Transcriptional Factor PU.1 Regulates Decidual C1q Expression in Early Pregnancy in Human

**DOI:** 10.3389/fimmu.2015.00053

**Published:** 2015-02-16

**Authors:** Shanmuga Priyaa Madhukaran, Uday Kishore, Kaiser Jamil, Boon Heng Dennis Teo, Mahesh Choolani, Jinhua Lu

**Affiliations:** ^1^Department of Microbiology, Yong Loo Lin School of Medicine, National University of Singapore, Singapore, Singapore; ^2^Centre for Biotechnology and Bioinformatics, School of Life Sciences, Jawaharlal Nehru Institute for Advanced Studies, Secunderabad, India; ^3^Centre for Infection, Immunity and Disease Mechanisms, College of Health and Life Sciences, Brunel University London, Uxbridge, UK; ^4^Division of Maternal-Fetal Medicine, Department of Obstetrics and Gynaecology, Yong Loo Lin School of Medicine, National University of Singapore and National University Health System, Singapore, Singapore

**Keywords:** C1q, decidua, pregnancy, trophoblast, stromal cells, pregnancy, implantation, transcription factor

## Abstract

C1q is the first recognition subcomponent of the complement classical pathway, which in addition to being synthesized in the liver, is also expressed by macrophages and dendritic cells (DCs). Trophoblast invasion during early placentation results in accumulation of debris that triggers the complement system. Hence, both early and late components of the classical pathway are widely distributed in the placenta and decidua. In addition, C1q has recently been shown to significantly contribute to feto-maternal tolerance, trophoblast migration, and spiral artery remodeling, although the exact mechanism remains unknown. Pregnancy in mice, genetically deficient in C1q, mirrors symptoms similar to that of human preeclampsia. Thus, regulated complement activation has been proposed as an essential requirement for normal successful pregnancy. Little is known about the molecular pathways that regulate C1q expression in pregnancy. PU.1, an Ets-family transcription factor, is required for the development of hematopoietic myeloid lineage immune cells, and its expression is tissue-specific. Recently, PU.1 has been shown to regulate C1q gene expression in DCs and macrophages. Here, we have examined if PU.1 transcription factor regulates decidual C1q expression. We used immune-histochemical analysis, PCR, and immunostaining to localize and study the gene expression of PU.1 transcription factor in early human decidua. PU.1 was highly expressed at gene and protein level in early human decidual cells including trophoblast and stromal cells. Surprisingly, nuclear as well as cytoplasmic PU.1 expression was observed. Decidual cells with predominantly nuclear PU.1 expression had higher C1q expression. It is likely that nuclear and cytoplasmic PU.1 localization has a role to play in early pregnancy via regulating C1q expression in the decidua during implantation.

## Introduction

Decidua plays a critical role in accepting the semi-allogenic fetus and protecting it from the mother’s immune system during pregnancy (1). Immunological cross-talk between the mother (stromal cells) and the fetus (trophoblasts) takes place in the decidua (2). Several immune and non-immune cells such as macrophages, dendritic cells (DCs), natural killer (NK) cells, T cells, stromal cells, and trophoblasts are involved in the maintenance of early pregnancy at the feto-maternal interface (3). Most of the decidual cells are hematopoietic cells derived from bone marrow (4–7). The process of lineage is tightly regulated by the transcription factors (8, 9). PU.1, an Ets-family transcription factor, was originally shown to be transcriptionally up-regulated in murine erythroleukemia following proviral integration of “spleen focus forming virus” (SFFV) (known as SP11) in humans (10, 11). PU.1 regulates the development and differentiation of myeloid lineage cells (macrophages, DCs, and neutrophils), B cells, NK cells, and T cells (12–15). In addition to being tissue-specific, PU.1 is also known to regulate hematopoiesis (16–18). Recently, PU.1 transcription factor has also been shown to regulate C1q gene expression in macrophages and DCs (19). The strong association between PU.1 and C1q expression raised the possibility that PU.1 may be responsible for C1q expression in the decidua during pregnancy.

C1q is the first recognition subcomponent of the complement classical pathway, which is expressed by the trophoblast and stromal cells in the decidua. Thus, C1q is considered to be involved in promoting interaction between endovascular trophoblast (ET) and endothelial cell. These fetal ET cells invading the decidua synthesize C1q as it differentiates into extravillous trophoblasts (EVT) (20, 21). Moreover, implantation sites in C1q knock-out mice contain reduced number of remodeled vessels and mirror the symptoms of human preeclampsia (22). Decidua from preeclampsia patients has a large number of unremodelled spiral arteries due to defective C1q expression (23). Therefore, C1q appears to regulate a range of functions of decidual cells that are critical for placental development and the absence of C1q leads to the onset of preeclampsia. However, the underlying mechanisms are not fully understood.

Here, we have analyzed human decidua during early pregnancy for the presence and distribution of C1q with the aim of determining whether PU.1 is involved in decidual regulation of C1q. We show that decidual cells express PU.1 transcriptional factors along with C1q. The present study suggests that localization of PU.1 may be critical for controlling decidual C1q expression.

## Materials and Methods

### Clinical samples

Decidual tissue samples were obtained from ten healthy women aged 20–35 years old who underwent elective vaginal termination of first trimester pregnancy (8–12 weeks). Informed verbal and written consent were obtained from all the subjects. The Domain Specific Review Board of National University Health System, Singapore approved the study. In each case, approximately 2–3 g of tissue were collected. All experiments were repeated at least minimum three times.

### Immunohistochemistry

Decidual tissues of approximately 1 cm^2^ were rinsed generously in phosphate buffered saline (PBS) to remove any blood clots. Tissues were fixed in 4% paraformaldehyde (PFA) at room temperature for 2–3 days and embedded in paraffin. Immunohistochemistry was carried out as described previously (24). Briefly, 5 μm thick sections of tissues were deparaffinized, and rehydrated in descending ethanol gradient (90, 70, 50, and 25% ethanol). The endogenous peroxidase activity was blocked using 0.3% hydrogen peroxide in dark and slides were boiled in sodium citrate buffer using microwave. The sections were permeabilized using 0.1% saponin in order to access the intracellular proteins. Non-specific binding of primary antibodies was blocked using 5% normal goat serum. Tissue sections were probed with rabbit anti-human PU.1 (H-135, Santa Cruz) and incubated overnight at 4°C. Following three washes in PBS, the sections were incubated for 2 h with anti-rabbit IgG secondary antibody conjugated with HRP in dark. Finally, substrate and chromogen (3,3′-diaminobenzidine DAB; Vector) were added to the slides and counterstained with hematoxylin. Primary antibody was replaced with rabbit IgG that served as negative control. The sections were viewed under a Leica DM3000 optical microscope Leica Microsystems), and images were taken using a digital camera (Leica Microsystems) on a computer hard drive.

### Isolation of decidual cells, decidual stromal cells

The methods were previously described by Singh et al. (24). Briefly, decidual cells were isolated by enzyme treatment and gradient centrifugation. The tissues were finely sliced into small pieces and washed using PBS with constant stirring to remove most of the remaining blood. The tissues were washed with PBS by centrifugation at 400 × *g* for 5 min. The tissues were suspended in RPMI-1640 medium at 37°C and incubated with collagenase type IV, for 1 h at 37°C. Later, centrifugation was carried out at 650 × *g* for 10 min. The supernatant containing released cells were discarded. The pellet was passed through 40 μm filters, washed three times in PBS by centrifugation, and left overnight to recover at 4°C with RPMI-1640 containing 10% v/v heat-inactivated FCS. Next day, the cells were collected after washing in PBS by centrifugation.

Decidual cells at the concentration of 1 × 10^6^ cells/ml were incubated in 12 well-plates (Nunc) with coverslips for 24 h in compete RPMI medium containing with 2% heat-inactivated FCS to allow stromal cells to adhere to the wells. After overnight incubation, supernatant containing non-adherent hematopoietic cells were discarded leaving 98% pure adherent stromal cells. Proliferating decidual stromal cells (DSCs) outgrew other possible contaminating cells, and thus, pure populations of cultured stromal cells were obtained. The supernatant was discarded; the cells were washed carefully, and then re-suspended in PBS for further analysis.

The concentration of decidual cells were adjusted to 0.050 × 10^4^ cells/200 μl using PBS. Slides were cytospun (700 rpm for 3 min) using cytocentrifuge (Shandon cytospin III) and air dried at room temperature. Slides were stored at room temperature overnight, or alternatively, used immediately for staining.

### Polymerase chain reaction

Total RNA was extracted from decidual cells and DSCs using Macherey-Nagel RNA isolation kit according to the manufacturer’s protocol. The concentration of the extracted RNA was determined by absorbance at 260 nm and the purity was estimated via A260/A280 ratio with Nanodrop spectrophotometer ND-1000 (Thermo Scientific). To evaluate the quality of RNA extracted, 1 μg of total RNA were reverse transcribed (Superscript II reverse Transcriptase kit, Invitrogen) and amplified (GoTaq PCR kit, Promega) with primers (custom-made by AIT biotech, Singapore) for the housekeeping gene GAPDH. Human macrophages cells, isolated as previously described by Cao et al. (25), were used as internal control. The PCR amplified products were separated on 1.5% agarose gel containing gel green along with 100-bp ladder (Fermentas) for visualization. GAPDH expression was used an endogenous reference gene and analyzed in parallel in all samples.

### Primers

For PU.1, the primers used were forward, 5′-AGAGCCATAGCGACCATTA-3′; and reverse, 5′-TATCGAGGACGTGCATCT-3′ (product, 162 bp). For GAPDH, the primers were forward, 5′-CGGAGTCAACGGATTTGGTCG-3′; and reverse, 5′-TCTCGCTCCTGGAAGATGGTGAT-3′ (product, 225 bp).

### Intracellular single immunofluorescence staining

Decidual cells on cytospin slides were fixed with 4% PFA. Cells were permeabilized with 0.1% saponin in PBS and then incubated with rabbit anti-human PU.1 (H-135) primary antibody in 5% BSA in PBS on ice, followed by staining with relevant goat-anti-rabbit FITC. Cells were washed in PBS, and then mounted using Vectashield with 4′, 6-diamidino-2-phenylindole (DAPI). The cellular localization of PU.1 with FITC was determined using Axiolmager Z1 fluorescent microscope. The images were captured and recorded with digital camera (Zeiss Axiocam MRc). Cells incubated with Isotype IgG were used as non-specific controls.

### Intracellular single immunofluorescence staining for confocal microscopy

Decidual cells on cytospin slides were fixed with 4% PFA. The cells were permeabilized with 0.1% saponin in PBS and then incubated with rabbit anti-human PU.1 (H-135) primary antibody in 5% BSA on ice, followed by staining with relevant goat-anti-rabbit IgG conjugated with Cy3. Cells were washed in PBS, and then mounted using Vectashield with DAPI. The cellular localization of goat-anti-rabbit Cy3 fluorescence showing the presence of PU.1 in decidual cells was determined using confocal microscope with 60× oil objective and the images were digitally recorded on a computer hard drive. Cells incubated with Isotype IgG were used as non-specific controls.

### Double immunofluorescence staining

#### Vimentin/PU.1

Decidual stromal cells on cytospin slides were incubated with mouse anti-human vimentin (1:150; Dako). After washing in PBS, slides were fixed in 4% PFA, and permeabilized in 0.1% saponin in PBS for 10 min. The slides were incubated with rabbit anti-human PU.1 H-135 (1:600; Santa Cruz). After washing with PBS, the slides were incubated with goat-anti-mouse FITC (3:100) and goat-anti-rabbit Cy3 (1:200) conjugates.

#### CK-7/PU.1

Decidual cells on cytospin slides were fixed with 4% PFA and permeabilized with 0.1% saponin in PBS for 10 min. The slides were incubated with mouse anti-human CK-7 (1:100; Abcam) and rabbit anti-human PU.1 H-135 (1:600, Santa Cruz). After washing with PBS, the slides were incubated with goat-anti-mouse Cy3 (1:200) and goat-anti-rabbit FITC (3:100) conjugates.

#### C1q/PU.1

Decidual cells on cytospin slides were fixed with 4% PFA and permeabilized with 0.1% saponin in PBS for 10 min. The slides were incubated with mouse anti-human C1q (1:200, Abcam) and rabbit anti-human PU.1 H-135 (1:600, Santa Cruz). After washing with PBS, the slides were incubated with goat-anti-mouse Cy3 (1:200) and goat-anti-rabbit FITC (3:100) conjugates. As controls, we analyzed the expression of the same in RAW cells using identical conditions.

All primary and secondary antibodies were diluted in 5% BSA and incubated 1 h in ice. The slides were finally mounted using Vectashield DAPI (Vectastain, Vector Laboratories). The cellular localizations of various proteins were examined using Zeiss Axiolmager Z1 fluorescent photomicroscope. The images were captured and recorded using digital camera (Zeiss Axiocam MRc). Cells incubated with Isotype IgG were used as non-specific controls.

## Results

### Immunohistochemical analysis of PU.1

**Figure 1:**
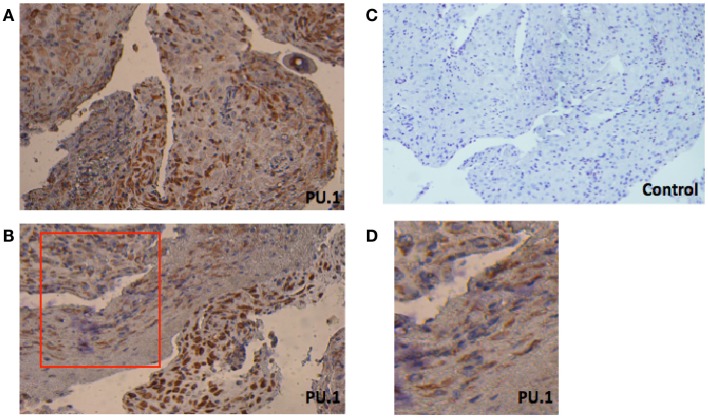
**Immunohistochemical localization of PU.1 in early pregnancy decidual tissue obtained from elective termination of pregnancy**. The tissues were stained by DAB and counterstained with hematoxylin, as described in Section “Materials and Methods.” The presence of PU.1 transcription factor is stained brown and hematoxylin in blue **(A,B)**. Control sections pre-incubated with rabbit IgG were uniformly negative **(C)**. The red box in **(B)** indicates enlarged image as seen in **(D)**. The experiment was performed using decidual tissues from five subjects. Original magnification 40×.

First, we investigated the presence of PU.1 in paraffin-embedded decidual sections obtained from women who underwent elective termination of pregnancy. As shown in Figures 1A,B, PU.1 immunostaining was intense in decidual tissues. PU.1 expression was consistent between decidual samples (case to case), but varied field to field. In few areas, weak and diffused staining was observed (Figure 1D). Notably, some cells did not stain for PU.1 suggesting that not all decidual cells express PU.1. We analyzed the expression of PU.1 in decidua obtained from five different subjects. Immunoreactivity was absent in control slides treated with rabbit IgG. Taken together, these results suggested that not all, but some, decidual cells synthesize PU.1 transcription factor.

### Expression of PU.1 in early human decidua analyzed by RT-PCR

**Figure 2:**
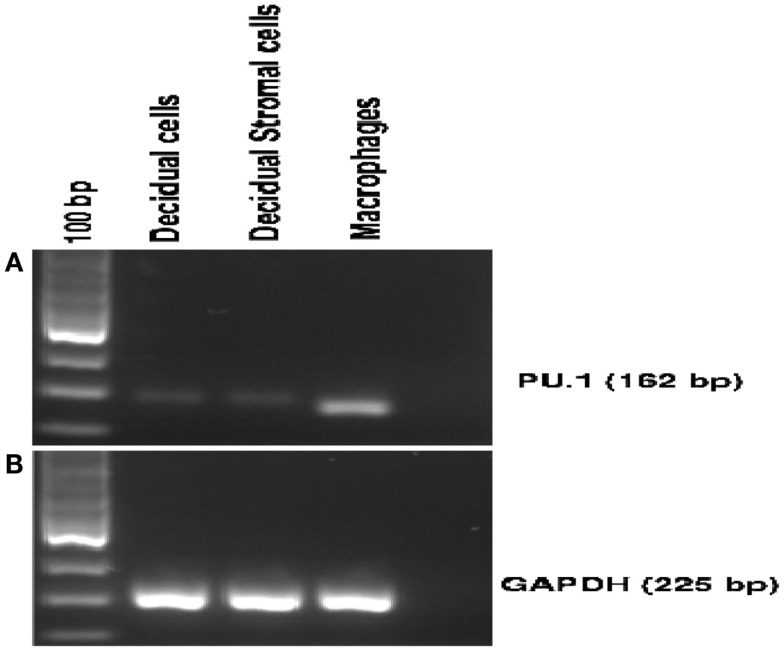
**Expression of PU.1 transcription factor by human first trimester decidua**. Decidual cells and decidual stromal cells isolated from human decidua were analyzed for PU.1 (162 bp) expression. For RT-PCR, 1 μg of total RNA isolated from decidual cells, decidual stromal cells, and human macrophages was used as a template. PU.1 cDNA from decidual cells, decidual stromal cells, and human macrophages were amplified using specific primer set mentioned in Section “Materials and Methods” **(A)**. GAPDH was amplified to serve as an internal control **(B)**. Human macrophage cells served as a positive control **(A)**. All four decidual samples were positive for PU.1 expression.

To further confirm the specificity of the antibody used in immunohistochemistry, we examined the expression of PU.1 mRNA in early human decidua. Using a fragment of 162 bp corresponding to human PU.1 proto-oncogene transcript variant 1 was amplified by RT-PCR. The PU.1 mRNA was easily detected in the decidual cells (Figure 2A). Since decidual cells expressed PU.1, we tested whether DSCs also expressed PU.1 mRNA. Thirty-five cycles of PCR amplification after the reverse transcriptase reaction yielded products derived from PU.1 mRNA in both decidual cells and DSCs (Figure 2A). The experiment was repeated with four different decidual samples. GAPDH primer pair was used as an internal control (Figure 2B) in addition to human macrophages cells that were used as a positive control (Figure 2A). The results confirmed the moderate-to-strong expression of PU.1 transcription factor at transcriptional as well as and translational level in decidual cells and DSCs.

### Cellular localization of PU.1 transcription factor in early human decidua

**Figure 3:**
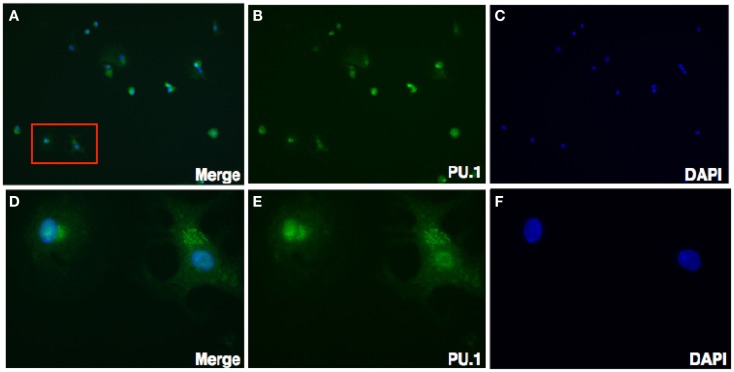
**Immunofluorescence staining for PU.1 expression in first trimester decidua**. Decidual cells cytospun were fixed, permeabilized, immunostained with rabbit anti-human PU.1 antibody, and then probed with goat-anti-rabbit FITC. After counterstaining with DAPI, analysis was carried out using Axiolmager Z1 fluorescent microscope. Decidual cells stained with DAPI, PU.1 and overlay at 40× magnifications **(A–C)**. Cells in red box **(A)** indicate the same at higher magnification, 100× oil immersion stained with DAPI, PU.1, and overlay in **(D–F)**.

To further characterize the cellular localization of PU.1 transcription factor, decidual cells were cytospun on coverslips, fixed, permeabilized, and immunostained with rabbit anti-human PU.1 (H-135) antibody. The cells were then mounted with DAPI. Transcription factors PU.1 has previously been localized in the nucleus (26). As expected, nuclear expression of PU.1 was detected in the decidual cells, although cytoplasmic staining of PU.1 was also observed (Figure 3). The immunostaining of decidual cells for PU.1 indicated the presence of PU.1 transcription factor in differential locations in various decidual cells.

### Examination of cellular localization of PU.1, transcription factor in early human decidua by confocal microscopy

**Figure 4:**
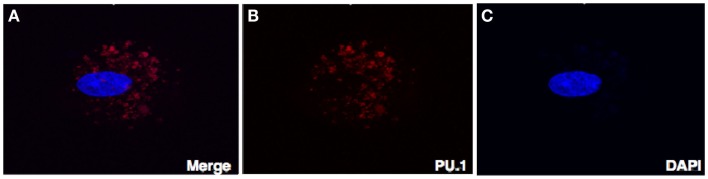
**Confocal microscopy of the decidual cells expressing PU.1 transcription factor in the cytoplasm and nucleus**. Decidual cells fixed, permeabilized, immunostained with rabbit anti-human PU.1 antibody and then probed with goat-anti-rabbit Cy3 **(B)**, counterstained with DAPI **(C)**, and analyzed by confocal microscope **(A)**. Original magnification 60×.

To rule out the possibility that localization of PU.1 in the cytoplasm is not due to goat-anti-mouse FITC coupled secondary antibody, we stained the decidual cells with rabbit anti-human PU.1 (H-135) antibody after permeabilization. Instead of FITC, goat-anti-rabbit Cy3 conjugate was used, counterstained with DAPI, and then analyzed by confocal microscope. As shown in Figure 4, PU.1 was localized in the cytoplasm of decidual cells. Although the reason for differential distribution of PU.1 in decidual cells is unclear and since PU.1 is well known for its role in hematopoiesis, it is likely that its expression is associated with different cell-cycle stages in early stages of pregnancy.

### Expression of PU.1 transcription factor by DSCs and trophoblasts

**Figure 5:**
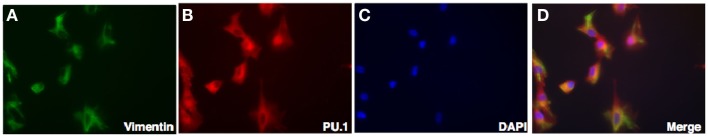
**Double immunofluorescence staining of PU.1 and stromal cells in first trimester decidua**. Pure population of stromal cells over coverslips was immunostained with mouse anti-human vimentin and rabbit anti-human PU.1 antibody. This was followed by probing with anti-mouse FITC and anti-rabbit Cy3, counterstained with DAPI and analyzed by Axiolmager Z1 fluorescent microscope at 40× magnifications **(A–D)**.

**Figure 6:**
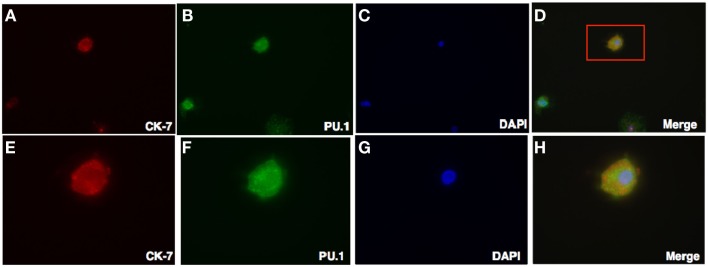
**Double Intracellular immunofluorescence staining of PU.1 and trophoblasts in first trimester decidua**. Decidual cells cytospun were fixed, permeabilized, and then immunostained with mouse anti-human CK-7 and rabbit anti-human PU.1 antibodies. This was then probed with goat-anti-mouse Cy3 and anti-rabbit FITC conjugates, counterstained with DAPI and analyzed by Axiolmager Z1 fluorescent microscope at 40× magnifications **(A–D)**. Cells in red box **(D)** indicate the same at higher magnification, 100× oil immersion stained with CK-7, PU.1, DAPI, and overlay in **(E–H)**.

Prompted by the PCR data, to gain insight into the intracellular localization of PU.1, we investigated stromal cells and trophoblasts by dual immunofluorescence against vimentin and cytokeratin-7. Majority of DSCs were found to express PU.1 either in the nucleus or in the cytoplasm (Figure 5). On the other hand, in trophoblasts cytoplasmic expression of PU.1 was observed. Remarkably, PU.1 positive staining was seen in very few trophoblasts (Figure 6). Thus, very few trophoblasts express PU.1, which mainly showed cytoplasmic staining. The presence of PU.1 with differential sub-cellular localization within the pure population of DSCs and trophoblasts confirms that PU.1 is definitely involved in the regulation of DSCs and trophoblasts. Furthermore, difference in the PU.1 staining pattern could possibly be due to its involvement in differentiation of DSCs and trophoblasts, thereby preparing for decidualization.

### C1q produced by decidual cells expressing nuclear PU.1

**Figure 7:**
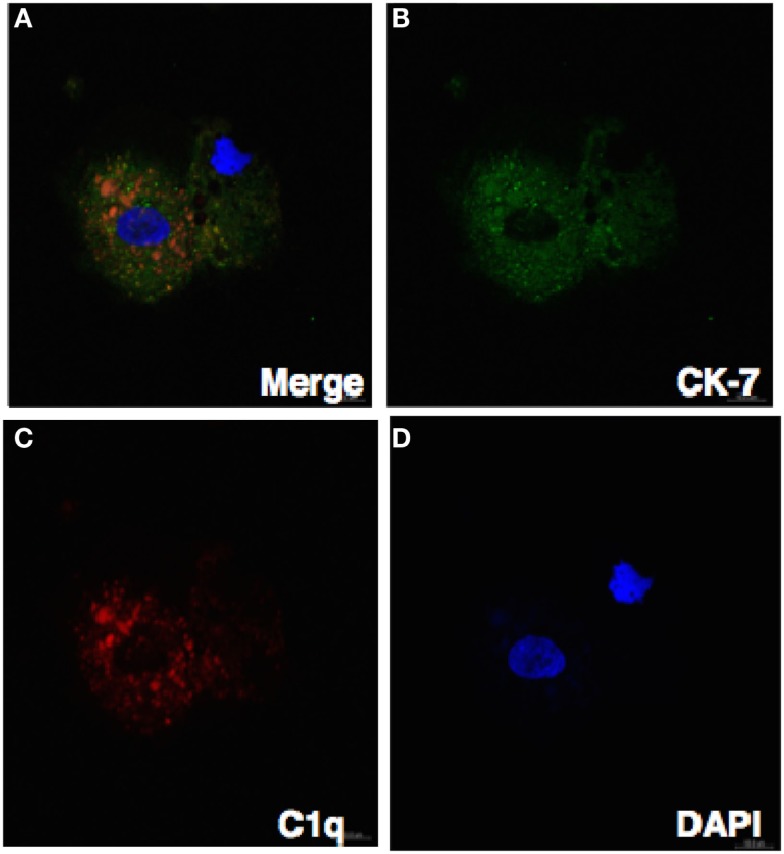
**Confocal image showing the trophoblast expressing C1q**. Decidual cells cytospun were fixed, permeabilized, immunostained with mouse anti-human CK-7 and goat-anti-human C1q antibodies, and then probed with donkey anti-mouse FITC and donkey anti-goat Cy3 conjugates, counterstained with DAPI and analyzed by confocal microscope **(A–D)**. Original magnification 60× oil immersion.

**Figure 8:**
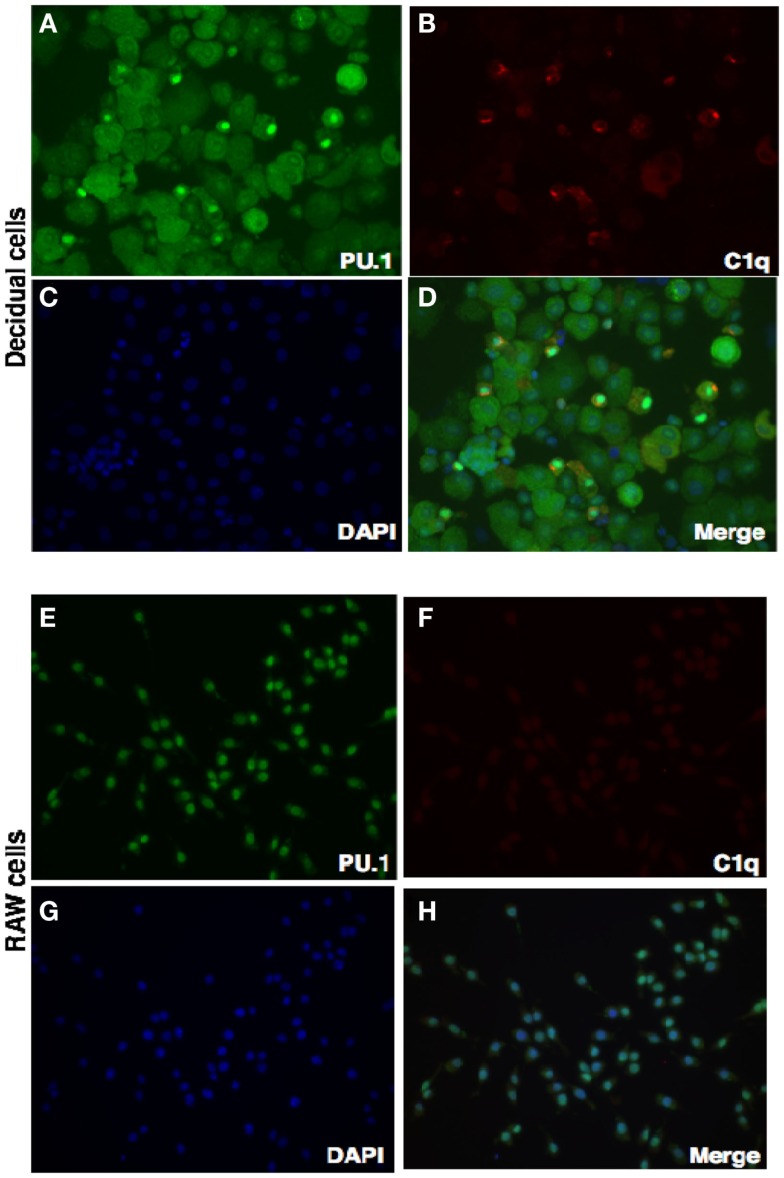
**Double intracellular immunofluorescence staining of PU.1 and C1q in first trimester decidua**. Decidual cells cytospun were fixed, permeabilized, and immunostained with mouse anti-human CK-7 and rabbit anti-human PU.1 antibodies. This was then probed with goat-anti-mouse Cy3 and anti-rabbit FITC conjugates, counterstained with DAPI and analyzed by Axiolmager Z1 fluorescent microscope **(A–D)**. RAW cells were used as controls treated and analyzed as decidual cells **(E–H)**. Original magnification for decidual cells and RAW cells are 20× magnifications.

We have shown via intracellular staining that DSCs and trophoblasts produce C1q (Figure 7), consistent with earlier studies (20, 21). To determine if PU.1 expressing cells also produced C1q, dual immunofluorescence staining was employed. Recent studies have indicated that PU.1 transcription factor regulates C1q expression in macrophages and DCs (19). Having found that PU.1 was widely distributed in the decidual cells (Figures 1 and 2), both PU.1 and C1q were detected in the decidual cells. Interestingly, decidual cells with significantly high levels of C1q expression showed PU.1 exclusively in the nucleus. They showed more intense staining in the nucleus but cytoplasmic staining was is also observed. (Figures 8A–D) when compared to RAW cells (Figures 8E–H). This indicates the close relationship between C1q and nuclear PU.1 gene expression.

## Discussion

In this study, we have investigated the relationship between PU.1 and C1q, and their likely regulation in early human decidua. We show that PU.1 is abundantly expressed in the decidua cells. To our knowledge, this is the first demonstration of the cellular localization of transcription factor PU.1 in the early human decidua. Nuclear PU.1 is likely to be a critical regulator of C1q expression in decidual cells based on the immunofluorescence staining pattern. PU.1 is an E26 transformation-specific (Ets) family transcription factor specifically involved in early and late stages of myeloid lineage cell differentiation (27). PU.1, known as SFFV proviral integration site 1 (SPi1) in humans, consists of an N-terminal transcriptional domain (100 amino acids) and a C-terminal DNA-binding domain (112 amino acids). Its gene is located on the shorter arm of chromosome 11p11.2 (28). The selected H-135 antibody raised against PU.1 recognizes amino acid 1-135 at the N-terminus of PU.1. Use of this antibody has previously established that PU.1 regulates C1q gene expression in macrophages and DCs (19).

Immunohistochemical analysis of paraffin-embedded first trimester decidua with rabbit anti-human PU.1 (H-135) showed a wide distribution of transcriptional factor PU.1. Overall, a strong immunoreactivity for PU.1 was observed (Figures 1A,B). To further validate this, we analyzed the PU.1 gene expression by RT-PCR in the first trimester decidua. Initially, we performed RT-PCR with decidual cells that suggested local synthesis of PU.1. Since DSCs are a key component of human decidua, we investigated the ability of DSCs to synthesize and secrete PU.1 (Figure 2A). Analysis of stromal cells also revealed the presence of PU.1, suggesting that decidual cells, in particular DSCs, expressed PU.1 at mRNA and protein level.

Next, we characterized the sub-cellular localization of PU.1 in decidual cells. Isolated decidual cells were cytospun, fixed, permeabilized, and immunostained with rabbit anti-human PU.1 (H-135) antibody. Surprisingly, the staining of the decidual cells with PU.1 indicated its differential locations among different cells within the decidua. Certain cells showed PU.1 expression in the nucleus whereas others showed immunofluorescence staining in the cytosol; some cells did not show PU.1 expression at all (Figure 3). This result was unexpected as the localization of transcription factor is usually within the nucleus. Because PU.1 was observed in the cytoplasm, we wanted to verify our speculation that the cellular localization of PU.1 was not due to the FITC conjugated secondary antibody. Therefore, we analyzed the cellular localization of PU.1 with Cy3 conjugated secondary antibody using confocal microscopy (Figures 4A–C). The observations from immunofluorescence staining by Axiolmager Z1 fluorescent and confocal microscopy demonstrated that PU.1 expression was present in a restrictive manner at specific locations within the decidual cells. Further studies on cellular localization of PU.1 in decidua will allow a better understanding of the functional significance of PU.1 in pregnancy.

To study the cell-type specific cellular localization of PU.1 in the first trimester decidua, stromal cells, and trophoblasts were stained with PU.1 and their respective markers vimentin and cytokeratin-7. Both stromal cells and trophoblasts were capable of synthesizing PU.1. Notably, all stromal cells were PU.1 positive (Figure 5) while very few trophoblasts revealed PU.1 expression (data not shown). The detection of increased nuclear PU.1 expression in stromal cells when compared to trophoblasts is puzzling. Furthermore, we observed that the PU.1 is expressed to a significantly higher level in the stromal cells than in the trophoblasts from first trimester decidua (Figures 5 and 6). Decidual leukocytes are proposed to trigger the endometrial stromal cell differentiation that encourages invasion of trophoblast by secreting IL-17 (29, 30). This cross-talk between stromal cells and trophoblasts is thus considered to regulate the immune milieu at feto-maternal interface for successful implantation.

To the best of our knowledge, this is the first study that addresses the expression of PU.1 in early human decidua. PU.1 not only regulates cellular process such as proliferation and differentiation but its relative expression levels are myeloid-specific regulating hematopoietic lineage commitment (13, 31–33). Our data strongly suggest that PU.1 is likely to be involved in stromal cell differentiation. The finding that few trophoblasts from first trimester decidua express PU.1 may pertain to special type of trophoblasts such as invasive EVT or syncytio-trophoblasts, which are further differentiating while invading the maternal decidua. Together, the differential expression patterns of PU.1 on stromal cells and trophoblasts reflect the differences in the requirement for specific functional roles in different cells within the decidua.

Previous studies have shown that first trimester decidual cells produce C1q and likely to play a role in trophoblast invasion and placental development (20, 21). Using immunofluorescence staining, we also show here the expression of C1q in both trophoblasts and stromal cells (Figure 7; Madhukaran et al., submitted). Next, we examined the ability of PU.1 to regulate C1q expression since PU.1 has previously been shown to regulate C1q gene expression in macrophages and DCs (19). To evaluate the contribution of PU.1 in decidual C1q expression, we immunostained decidual cells for PU.1 and C1q. Strong staining for C1q and PU.1 was observed in the decidual cells when compared to RAW cells (Figure 7). Moreover, we observed that PU.1 expression was confined to the nucleus in decidual cells that had significant increase in C1q expression (Figures 7A–D). The immunofluorescence staining data indicate, for the first time, the presence of nuclear PU.1 location in decidual cells expressing C1q. In contrast, cytoplasmic localization of PU.1 staining was also detected in cells with no or less C1q expression. The presence of nuclear PU.1 in decidual cells expressing C1q suggests that PU.1 may be regulating the production of decidual C1q. We have recently found high level of C1q expression in all stromal cells, while very few trophoblasts exhibit C1q expression (Madhukaran et al., submitted). It would be of interest to study the expression levels of both PU.1 and C1q in stromal cells and trophoblasts associated with normal pregnancy and preeclampsia in humans.

If PU.1 and C1q are normally expressed in early human decidua, their relative levels could regulate trophoblast and stromal cell specific lineage differentiation during early implantation. The association of PU.1 and C1q expression in first trimester human decidua and the exact role may be fundamental to our understanding of the lineage commitment of PU.1 that regulates C1q expression during normal pregnancy. Taken together, our data suggest that decidual cells transcribe and translate PU.1 and it is selectively localized in the stromal cells and trophoblasts. Additional studies aimed at understanding the PU.1 regulation of C1q gene expression in decidua during normal pregnancy are required. It also raises the possibility of understanding the functional implications of cytoplasmic PU.1 in early human decidua during implantation of normal as well as complicated pregnancies such as preeclampsia.

## Conflict of Interest Statement

The authors declare that the research was conducted in the absence of any commercial or financial relationships that could be construed as a potential conflict of interest.
